# Photothermally controlled ICG@ZIF-8/PLGA coating to modify the degradation behavior and biocompatibility of Zn-Li alloy for bone implants

**DOI:** 10.1093/rb/rbaf001

**Published:** 2025-01-06

**Authors:** Ting Zhang, Yameng Yu, Wei Yuan, Zeqi Ren, Yan Cheng, Shuilin Wu, Yufeng Zheng, Dandan Xia

**Affiliations:** Department of Dental Materials, Peking University School and Hospital of Stomatology, Beijing, 100081, China; National Center for Stomatology, Beijing, 100081, China; National Clinical Research Center for Oral Diseases, Beijing, 100081, China; National Engineering Research Center of Oral Biomaterials and Digital Medical Devices, Beijing, 100081, China; Beijing Key Laboratory of Digital Stomatology, Beijing, 100081, China; NHC Key Laboratory of Digital Stomatology, Beijing, 100081, China; NMPA Key Laboratory for Dental Materials, Beijing, 100081, China; School of Materials Science and Engineering, Peking University, Beijing, 100871, China; Department of Dental Materials, Peking University School and Hospital of Stomatology, Beijing, 100081, China; National Center for Stomatology, Beijing, 100081, China; National Clinical Research Center for Oral Diseases, Beijing, 100081, China; National Engineering Research Center of Oral Biomaterials and Digital Medical Devices, Beijing, 100081, China; Beijing Key Laboratory of Digital Stomatology, Beijing, 100081, China; NHC Key Laboratory of Digital Stomatology, Beijing, 100081, China; NMPA Key Laboratory for Dental Materials, Beijing, 100081, China; School of Materials Science and Engineering, Peking University, Beijing, 100871, China; School of Materials Science and Engineering, Peking University, Beijing, 100871, China; School of Materials Science and Engineering, Peking University, Beijing, 100871, China; School of Materials Science and Engineering, Peking University, Beijing, 100871, China; School of Materials Science and Engineering, Peking University, Beijing, 100871, China; Department of Dental Materials, Peking University School and Hospital of Stomatology, Beijing, 100081, China; National Center for Stomatology, Beijing, 100081, China; National Clinical Research Center for Oral Diseases, Beijing, 100081, China; National Engineering Research Center of Oral Biomaterials and Digital Medical Devices, Beijing, 100081, China; Beijing Key Laboratory of Digital Stomatology, Beijing, 100081, China; NHC Key Laboratory of Digital Stomatology, Beijing, 100081, China; NMPA Key Laboratory for Dental Materials, Beijing, 100081, China

**Keywords:** Zn-based alloys, ZIF-8, corrosion resistance, photoresponsive, surface modification

## Abstract

Biodegradable Zn alloy has recently gained attention for use in bone implants considering its biodegradability, attractive mechanical properties and bioactivity. However, excessive corrosion of Zn alloy at the early stage of implantation may cause severe cytotoxicity, resulting in insufficient osseointegration, which hinders the clinical use of Zn alloy. In this study, we designed a photothermally controlled degradative hybrid coating as a corrosion-protective barrier with the intention of preventing Zn ion burst release during the early stages of implantation and regaining the alloy’s corrosion advantage later on. The coating consists of zeolite imidazole skeleton-encapsulated indocyanine green core–shell-structured nanoparticles and polylactic coglycolic acid (ICG@ZIF-8/PLGA) on pristine Zn-0.8 (wt.%) Li (ZL) alloy. The electrochemical test results indicated that coating ZL with ICG@ZIF-8/PLGA can effectively reduce its corrosion current density (i_corr_) from 2.48 × 10^−5^ A·cm^−2^ to 2.10 × 10^−8^ A·cm^−2^. After near-infrared (NIR) light irradiation, ICG@ZIF-8 heated PLGA coating to reach Tg, causing the coating to degrade and the i_corr_ of the coated ZL alloy decreased to 2.50 × 10^−7^ A·cm^−2^, thus restoring corrosion advantage. Both *in vitro* and *in vivo* investigations showed that the coated ZL alloy had acceptable biocompatibility. Overall, the developed photothermally controlled coating improved the Zn alloy’s resistance to corrosion and allowed for the adjustment of the Zn alloy’s degradation rate through 808-nm NIR light irradiation.

## Graphical Abstract

**Figure:**
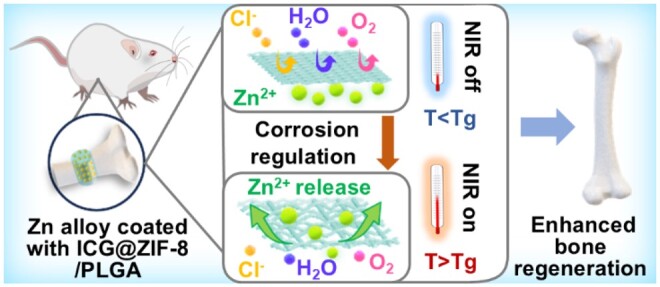

## Introduction

Biodegradable zinc (Zn) metals have received a lot of attention in orthopedic fields [1, 2], due to its intermediate corrosion potential between Mg and Fe [3–5]. Among various Zn alloys, the Zn-lithium alloy (Zn-Li) stands out as a highly promising candidate for orthopedic implants, as it is expected to maintain the necessary mechanical strength throughout the fracture repair process [3, 6–8]. Zn^2+^ plays a crucial role in bone tissue development, maintaining equilibrium, the synthesis of collagen and the process of mineralization [9]. The release of Zn^2+^ may exhibit cytotoxicity since cell response to Zn^2+^ is dose dependent, and a low concentration of Zn^2+^ promotes cell activity, whereas excessive release of Zn^2+^ can hinder cell adhesion and migration [4, 10–12]. It should be noted that a significant release of Zn^2+^ during the early phases of implantation in the femoral condyles of rats may lead to poor osseointegration [10]. The development of surface modification techniques that grant Zn alloy manageable degradation characteristics and optimal properties for bone integration is eagerly anticipated [2, 4, 11–13].

A range of surface modification techniques have been used to create various coatings on Zn alloy. In addition to conventional techniques like micro-arc oxidation (MAO) [14], protective layers like polycaprolactone (PCL) and polylatic acid (PLA) were also used to improve corrosion behavior [15]. Chemical deposition coating has attracted increasing attentions due to its biological traits in the recent years [11, 16, 17]. Among the representative coating materials, polylactic acid-glycolic acid (PLGA) exhibited advantages of drug delivery capabilities and customized degradation rates. More importantly, due to its semi-crystalline, PLGA has an obvious barrier effect on the substrate; therefore, PLGA is also utilized to increase the degradable metals’ resistance to corrosion [18]. PLGA-based self-healing systems have been developed to reduce corrosion in magnesium alloys [19–22]. However, PLGA coating is mostly degraded in a passive form. How to actively regulate the degradation of PLGA coating in order to control the corrosion behavior of Zn alloy remains to be studied.

Smart coatings can control the rate at which degradable metals corrode by reacting to a variety of environmental stimuli, including light, sound and electricity. These external stimuli can be employed as precise drive switches [23–25]. Photothermal stimulation represents a reliable method and has been widely studied [26]. Near-infrared (NIR) light irradiation is widely utilized for noninvasive treatment in biomedicine, primarily due to its excellent light irradiation transmission through biological tissues and high spatio-temporal accuracy [2, 27, 28]. Indocyanine green (ICG) is a popular NIR organic dye with outstanding photothermal conversion efficiency, high biocompatibility and has the ability to produce local heat when exposed to 808-nm NIR light [29], superior photothermal conversion efficiency. Therefore, ICG can be considered an effective heating agent to facilitate Tg attainment by PLGA. However, poor aqueous stability and rapid *in vivo* clearance rate impede the clinical application of ICG [30]. Recent advances in nanotechnology have provided new opportunities for prolonged circulation of photosensitizers [30]. Metal-organic frameworks can preserve the stability and activity of these guest molecules and have a high loading capacity for biomolecules [31–34]. Zeolite imidazole framework (ZIF-8) has garnered extensive attention due to its favorable biocompatibility and exceptional drug encapsulation capabilities [27]. The sulfonic acid group in ICG produces a strong intermolecular binding force with ZIF-8, which can be used as an encapsulation matrix for ICG. By constructing a core–shell structure, the release of ICG is strictly confined by ZlF-8, which can effectively prevent the aggregation of ICG and enhance its aqueous stability.

Herein, we described a biodegradable hybrid ICG@ZIF-8/PLGA coating to modify the degradation of Zn-0.8Li alloy (ZL) by 808-nm NIR irradiation. PLGA enhanced the ZL alloy’s biocompatibility and decreased its exposure to liquid environments, while ICG in the core provided excellent photothermal effects and the ZIF-8 shell enhanced ICG’s stability. ICG@ZIF-8 was expected to endow the hybrid coating with durable NIR responsiveness, high NIR absorption capacity and photothermal properties, thereby facilitating the degradation of the PLGA coating through photothermal response in the later stage of implantation. ZL coated with the ICG@ZIF-8/PLGA hybrid coating showed good biocompatibility based on *in vitro* and *in vivo* evaluations. Additionally, the photothermal response coating increased the Zn alloy’s resistance to corrosion and allowed for the use of 808-nm NIR laser irradiation to regulate the degradation of the coated ZL alloy. This coating presents the possibility of more widespread uses for Zn implants.

## Material and methods

### Reagents

Alfa Aesar Country supplied Zn(NO_3_)·6H_2_O, Aladdin supplied Hmim (2-methylimidazole), HARVEYBIO supplied ICG, and Engineering For Life supplied PLGA, which had a mean molecular weight of 120 000, with a co-polymer composition of 85–15, and a viscosity measurement of 0.61. Cast Zn-0.8Li (wt. %) (ZL) binary alloy was fabricated by Rare Earthand Alloy Research Institute, Hunan Province, China. After cutting the ZL alloy into Ø10 mm × 1 mm plates and Ø2 mm × 6 mm bars, they were ground using SiC sandpapers with varying grits (400, 800, and 2000) in succession. These substrates were then ultrasonically cleaned for 15 minutes in ethanol and acetone, respectively. Thirty minutes prior to testing, the ZL alloy was removed from its ethanol storage and dried using N_2_.

### ZIF-8 and ICG@ZIF-8 *in situ* growing on ZL alloy plate surfaces

Under ambient circumstances, the preparation of the ZIF-8 synthesis mixture involved 2.27 g of 2-methylimidazole, and 0.11 g of Zn(NO_3_)_2_·6H_2_O in water of 40 mL. The ZL alloy plate was then immediately submerged in the ZIF-8 precursor solution, and subsequently, the ZL alloy plate was promptly dipped into the ZIF-8 precursor liquid and allowed to soak for 36 hours. The resulting composite membrane was vacuum-dried for 12 hours after being cleaned with ethanol and water. We took the following actions in the dark. The ICG@ZIF-8 was prepared under ambient conditions using 0.11 g Zn(NO_3_)·6H_2_O, 50 mg ICG, and 2.27 g 2-methylimidazole dissolved in water. Next, for 36 hours at room temperature, the ZL alloy plate was submerged right away in the ICG@ZIF-8 precursor solution. The resulting composite membrane was vacuum-dried for 12 hours after being cleaned with ethanol and water.

### Synthesis of PLGA coating

PLGA powder was dissolved in chloroform solvent, which had a 4% (w/v) PLGA concentration. The solvent casting technique was used. The ZL alloy’s surface, which had previously been coated with ZIF-8 or ICG@ZIF-8, was carefully dispensed with an 80 μL aliquot of the PLGA solution. After that, the samples were dried for later use at 37°C.

### Surface characterization of composite membranes

Scanning electron microscopy (SEM, S-4800, Hitachi, Japan) was employed for surface investigation of the surface coating and bare ZL alloy morphology. SEM was also used to determine the ZL/ICG@ZIF-8/PLGA sample’s thickness, and energy spectra were used to determine the cross- section’s element distribution. Surface phase analysis was conducted using X-ray diffraction (XRD, Rigaku DMAX 2400, Japan), scanning from 10° to 90° in 2θ at a rate of 4° per minute. The surface chemical makeup of the samples was examined using Fourier transform infrared spectroscopy (FT-IR, Nicolet is50, USA). To determine the melting temperature of the PLGA sample, differential scanning calorimetry (DSC, Q2000, USA) was employed. The absorbance spectra were obtained using the ultraviolet–visible (UV–Vis) NIR (UV-3600PLUS, Japan).

### Electrochemical corrosion tests

A traditional three-electrode setup was used to conduct electrochemical measurements. The working electrode for electrochemical testing was a platinum electrode, and a reference electrode was made of saturated calomel. Using an electrochemical station (Autolab, Metrohm, Switzerland), the test procedure was carried out in Hanks’ solution (pH 7.4, Na_2_HPO_4_·12H_2_O 0.06 g·L^−1^, KH_2_PO_4_ 0.06 g·L^−1^, MgSO_4_·7H_2_O 0.06 g·L^−1^, MgCl_2_·6H_2_O 0.10 g·L^−1^, CaCl_2_ 0.14 g·L^−1^, KCl 0.40 g·L^−1^, NaHCO_3_ 0.35 g·L^−1^, glucose 1.00 g·L^−1^ and NaCl 8.00 g·L^−1^). Each specimen was subjected to a 3600-second monitoring period for open circuit potential. Electrochemical impedance spectroscopy measurements were taken with an AC perturbation of 10 mV, spanning a frequency spectrum from 10^5^ to 10^−2 ^Hz. Additionally, the potentiodynamic polarization curve, also known as the Tafel plot, was recorded at a scan rate of 1 mV per second.

### NIR light irradiation tests

A forward-looking infrared (FLIR) thermal camera (E50, Teledyne FLIR, USA) was used to measure the samples’ photothermal characteristics. The samples’ photothermal heating and cycle curves were assessed when subjected to 808-nm NIR light (0.3 W·cm^−2^). The surface morphology of the samples following NIR light irradiation was investigated using SEM.

#### In vitro cytocompatibility studies

For the cytotoxicity tests, ISO 10993-12:2020 was followed. To evaluate the cytotoxicity of the coating, we established three experimental groups: untreated ZL alloy (control group), ZL/ICG@ZIF-8/PLGA coated alloy and ZL/ICG@ZIF-8/PLGA coated alloy with 808-nm NIR light illumination. The disinfected specimens were transferred to sterile dishes and soaked in a serum-enriched medium with a volume of 1.25 mL for every square centimeter of the sample’s surface. They were then incubated for a day under the same environmental settings. MC3T3-E1 osteoblasts were incubated in medium for 24 hours, after which their medium was replaced with the sample extract. In the experimental group receiving light treatment, each sample was subjected to 20 minutes of NIR light irradiation. Post-incubation periods of 1, 2 and 3 days, the used medium was removed and replaced with an updated mixture of cell culture medium that included an addition of 10% Cell Counting Kit-8 (CCK-8, HY-K0301, MedChemExpress, USA). The culture was then allowed to proceed for an additional 2 hours. Microplate reader (model 1681130, Bio-Rad, USA) was used to record the absorbance at a wavelength of 450 nm to get the optical density. For each time point, the analysis was conducted on five replicate samples to ensure statistical reliability.

To investigate the impact of sample extracts on MC3T3-E1, the cells were plated in a 24-well plate at a uniform density and incubated for a 24-hour period. After that, the sample extracts were replaced and then cultivated for a further 24 hours. Post-incubation, the cells were subjected to three rinses with phosphate-buffered saline at pH 7.4, then stabilized using 4% paraformaldehyde for a 10-minute duration and subsequently permeabilized for 10 minutes with 0.5% Triton X-100. Following this, the actin filaments were labeled with 1.0% FITC-phalloidin (product P5282 from Solarbio, China) for a 1.5-hour duration at ambient temperature. Concurrently, the nuclei were counterstained with 4,6-diamino-2-phenylindole (DAPI, supplied by Solarbio, China) for a 10-minute period. After staining, the samples underwent three rinses. The fabricated samples were investigated using a confocal laser scanning microscope, using excitation at wavelengths of 488 nm and 405 nm.

The movement of cells was evaluated through a wound-healing assay. For this assay, cells of MC3T3-E1 were cultivated until 90% fusion was achieved. A 200-μL pipette tip was then used to produce a scratch. Following this, serum-free medium mixed with different material extracts was used to culture the cells for 0, 12 and 24 hours. Afterward, the scratch width was evaluated using an optical microscope (BX-51, Olympus, Japan).

#### In vivo trails

The osteogenic potential of the coated alloys was assessed utilizing a bone-defect animal model. In this study, ZL/CG@ZIF-8/PLGA served as the experimental group, while ZL acted as the control group. Each group was further divided either to receive NIR light irradiation or to remain without light exposure, in order to evaluate the impact of irradiation on osteogenesis. The Peking University Biomedical Ethics Committee’s Experimental Animal Welfare Ethics Branch gave its approval to the animal trials (Approval Number: LA2022029). Twenty-four healthy Sprague–Dawley rats (male, 10 weeks old, weighing 250–270 g) were placed in eight cages at random and allowed to acclimate for a week in the specific pathogen free (SPF) laboratory. The rats were randomly divided into four groups as follows: (i) ZL alloy without NIR light irradiation (D), (ii) ZL alloy with NIR light irradiation (L), (iii) ZL/CG@ZIF-8/PLGA without NIR light irradiation (D) and (iv) ZL/CG@ZIF-8/PLGA with NIR light irradiation (L) (*n* = 6).

The rats were given 50 mg/kg of 1% pentobarbital sodium intraperitoneally to induce unconsciousness in order to reduce their level of suffering. The bilateral cavities with a diameter of 2 mm and a depth of 6 mm in the distal area of the rats’ femurs were drilled using a dental implant tool model SI-923 from W&H Dental werk Burmoos GmbH, Austria. Subsequently, ZL alloys of the same dimensions were inserted into the prepared holes, either with or without additional coating. The experimental group exposed to light therapy received a 20-minute irradiation of NIR light on the implant surface. There were no fatalities among the animals during or following the surgical procedure, and all experimental units or datapoints were included in the analysis. All rats were kept in individually sterilized cages for a period of 2 months, after which they were humanely euthanized using an overdose of sodium pentobarbital. The femurs, lungs, hearts, livers, kidneys and spleens from the rats were excised and preserved in a 10% neutral formalin solution for a day at ambient temperature. SEM was utilized to assess the corrosion patterns of the implants cross-sectionally. Hematoxylin and eosin (H&E) staining was applied to the paraffin-embedded sections, which were then examined using a microscope (Olympus, Germany). Additionally, histopathological assessments of the primary organs were conducted to gauge the toxicity of the test samples *in vivo*, including ZL alloy, ICG@ZIF-8/PLGA-coated ZL alloy, and those treated with NIR radiation.

### Statistical analysis

Data were shown as mean ± standard deviation. Group differences were analyzed using one-way ANOVA and Tukey’s test for post-hoc comparisons. Statistical significance denoted by * for *P* < 0.05, ** for *P* < 0.01, *** for *P* < 0.001 and **** for *P* < 0.0001; ns indicates *P* > 0.05 (no significance). *In vitro n* = 3, *in vivo n* = 6.

## Results

### Preparation and characterization of hybrid coatings

**Figure 1.**
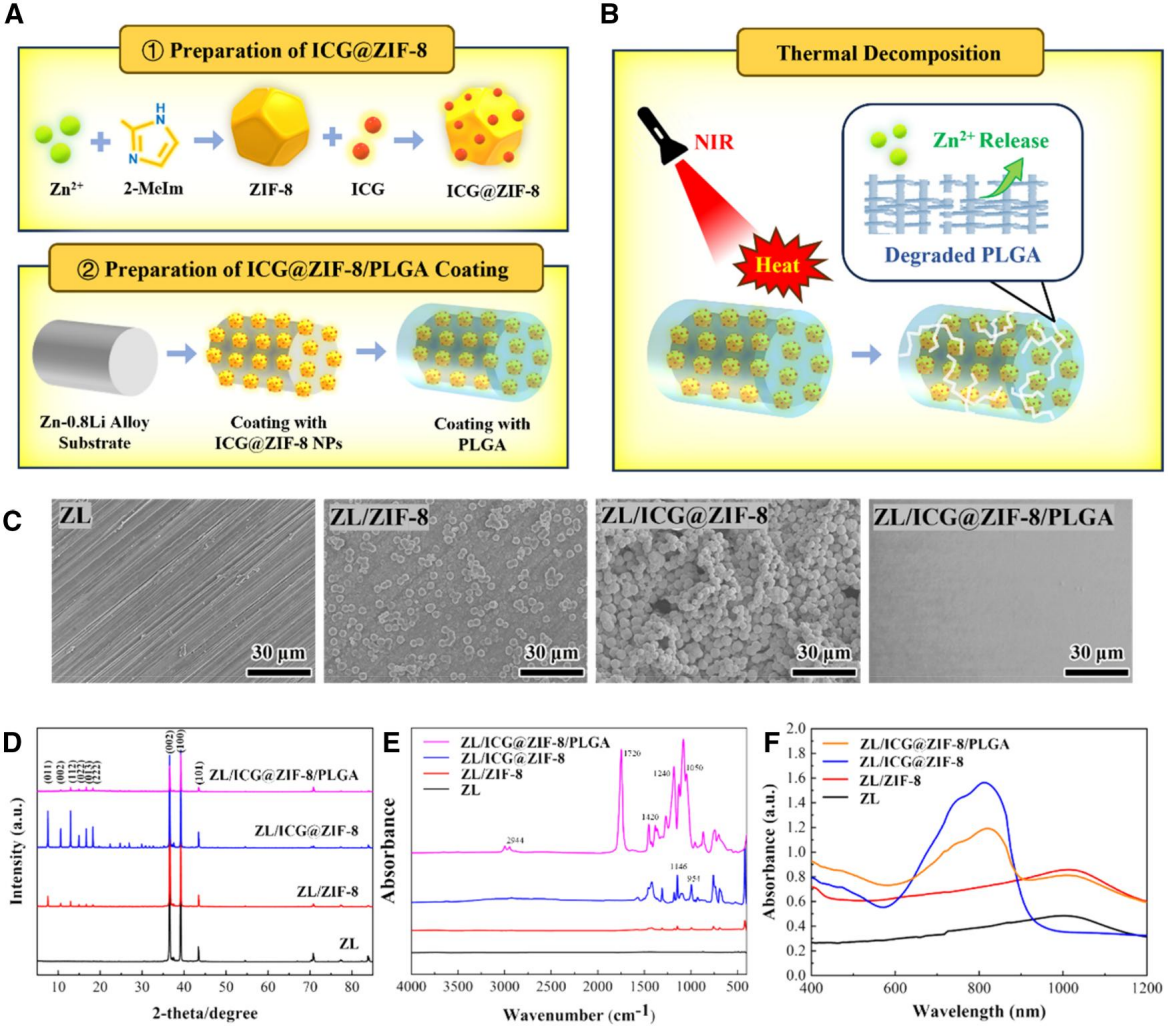
(**A**) Preparation of lCG@ZlF-8, (**B**) schematic representation of the ICG@ZIF-8/PLGA coating synthesis process on ZL alloy, (**C**) surface morphologies of different samples, (**D**) XRD pattern of different samples, (**E**) FTIR spectral analysis and (**F**) UV–Vis–NIR spectral absorption of different samples.

Figure 1A–B showed the schematic representation of the thermal decomposition process and the synthesis routine of ICG@ZIF-8/PLGA coating. Figure 1C showed the surface morphologies of different samples. The ZL alloy’s surface had a few scratches that resulted from mechanical polishing. Following ZIF-8 or ICG@ZIF-8 coating, the samples displayed comparable surface morphologies, sizes and forms. It was possible to see distinct ZIF-8 and ICG@ZIF-8 nanoparticles with hexagonal facets [35]. In contrast, ICG@ZIF-8/PLGA coating exhibited uniform surface morphology without scratches. And the thickness of ZL/ICG@ZIF-8/PLGA sample was about 100 µm (Supplementary Appendix Figure 1). XRD spectrums (Figure 1D) showed that the crystallization properties of ICG@ZIF-8 composites were unaffected by the addition of ICG to the ZIF-8 frame, and a significant diffraction peak at 2θ value between 5° and 20° remained, comparable to that of pure ZIF-8. According to FT-IR spectrum in Figure 1E, the ZL/ICG@ZIF-8 spectrum displayed absorption peaks at 1310, 1146 and 954 cm-1, corresponding to the vibrational modes of C-N stretching, C-H bending and the bending of the imidazole ring within the 2-methylimidazole connector, respectively. The FT-IR spectrum of the ICG@ZIF-8/PLGA coating revealed three distinct peaks at 2944, 1720 and 1240 cm^-1^, which are associated with the asymmetric stretching of CH_2_, the stretching of C=O and the asymmetric stretching of C-O-C bonds, respectively. Spectral peaks occurring at 1050 and 1420 cm^−1^ corresponded to asymmetric vinyl stretching and C=C stretching, respectively [36, 37]. Pure ZL alloy had no obvious absorption peak. FT-IR spectral analysis verified the incorporation of ZIF-8 and ICG within the freshly synthesized ZL/ICG@ZIF-8/PLGA coatings. Besides, UV–Vis (Figure 1F) revealed that the ZL/ICG@ZIF-8 and ZL/ICG@ZIF-8/PLGA samples displayed pronounced absorbance within the NIR spectrum ranging from 600 to 900 nm, confirming the incorporation of ICG [38]. Conversely, no significant absorption was observed for the ZL alloy and ZL/ZIF-8. The results indicated that a successful coating layer was deposited onto the ZL alloy’s surface.

### Photothermal performance of hybrid coatings

**Figure 2.**
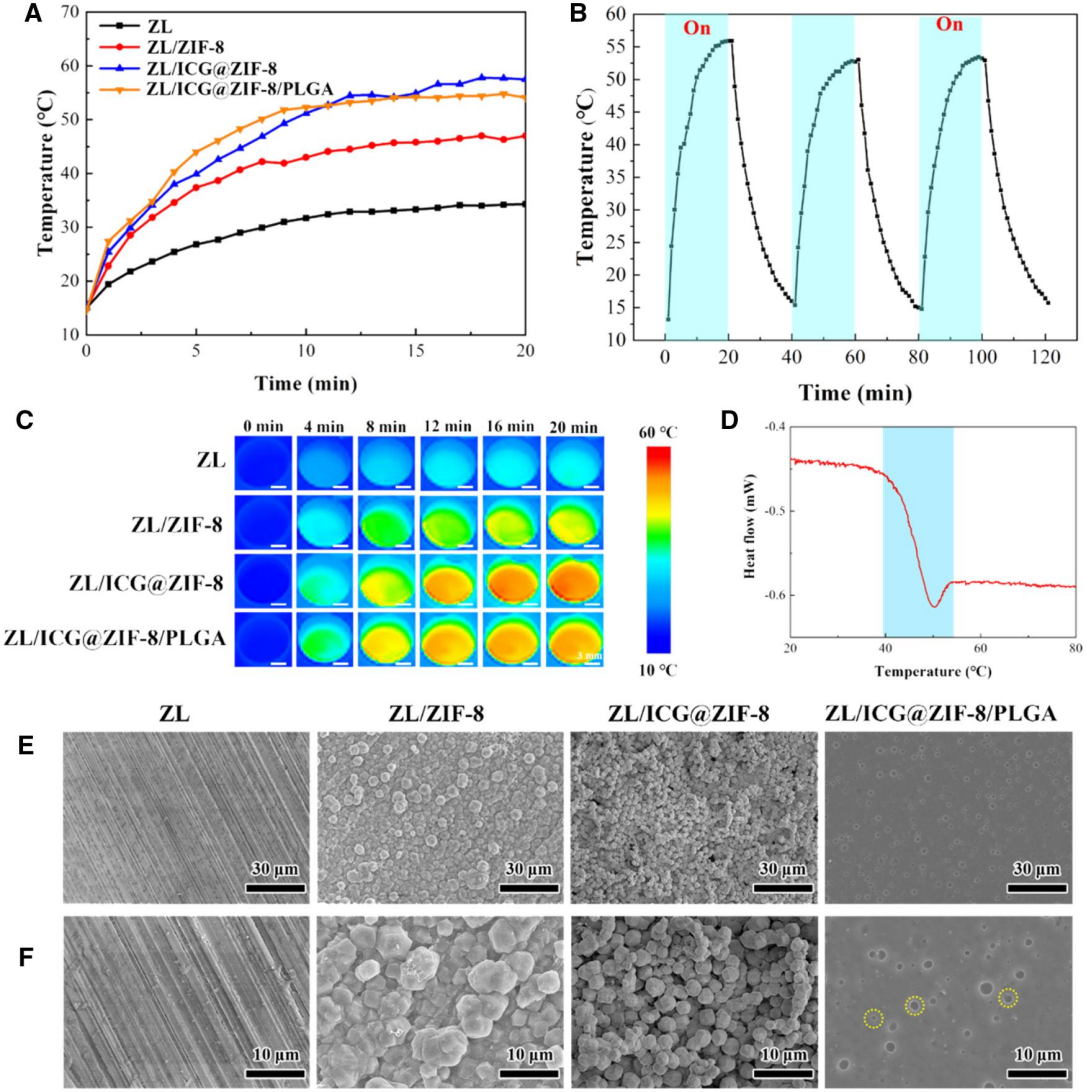
(**A**) Photothermal heating curves of different samples, (**B**) thermal fluctuation profiles during successive cyclic light exposure of ZL/ICG@ZIF-8/PLGA coatings, (**C**) infrared thermography of different samples exposed to 808-nm NIR light (0.3 W·cm^−2^), (**D**) DSC curves of components of PLGA in coatings, (**E**) Surface morphologies of different samples following a 20-minute exposure to NIR light with a 0.3 W·cm^−2^ power density, (**F**) a larger view of figure (**E**).

Figure 2 demonstrated the photothermal behavior of various samples subjected to NIR light (0.3 W·cm^−2^). Figure 2A displayed the temperature profiles, which were captured at 60-second intervals, together with the cyclic light irradiation curves. Figure 2B illustrated that after 20 minutes of laser light irradiation, the thermal images of ZL/ICG@ZIF-8 and ZL/ICG@ZIF-8/PLGA exhibited quite arresting temperature increment from 15°C to 55°C. Meanwhile, the thermal images of ZL alloy and ZIF-8 only rose from 15°C to 34°C and 46°C, respectively, manifesting the photothermal activity of ICG embedded in the coating [22]. The stability of photothermal response and the durability of on–off laser cycling for the ICG@ZIF-8/PLGA coating were examined (Figure 2B). Exposure to NIR light led to a swift temperature rise in the ICG@ZIF-8/PLGA-coated samples, peaking at around 55°C in a span of 10 minutes. The laser switching period of ICG@ZIF-8/PLGA coating showed that the photothermal effect had no obvious decreasing trend, indicating that the photothermal performance of the coating did not decrease significantly after repetition. The change in surface temperature over time under NIR light exposure was also visually captured through photothermal imaging (Figure 2C), aligning closely with the data presented in Figure 2A.

Figure 2D presented the DSC thermal curves. These curves revealed the heat absorption peaks of PLGA. The glass transition temperature (Tg) was identified using the mid-point method. Notably, the Tg was lower than the peak photothermal temperature of 55°C, as indicated in Figure 2A. The findings suggested that 808-nm NIR light can activate ICG to produce heat, leading to the deformation of PLGA and the fragmentation of the chain. Thus, the electrolyte could damage the ZL alloy by penetrating the composite covering. Figure 2E–F depicted the surface characteristics of various specimens following exposure to NIR radiation. The surface textures of the ZL alloy, ZL/ZIF-8 and ZL/ICG@ZIF-8 composites exhibited minimal alterations when subjected to NIR radiation, in contrast to their unirradiated states. However, ZL/ICG@ZIF-8/PLGA under NIR light irradiation was seriously corroded, and there were some corroded pores (represented by yellow circles) in the coating. Obviously, there were some defects and thermal expansion damage on the surface (Figure 2F), indicating that the composite coating was damaged by NIR irradiation. Additionally, these defects made it possible for the electrolyte to penetrate the coating, make contact with the substrate, and react with the ZL alloy.

### Electrochemical measurements

**Figure 3.**
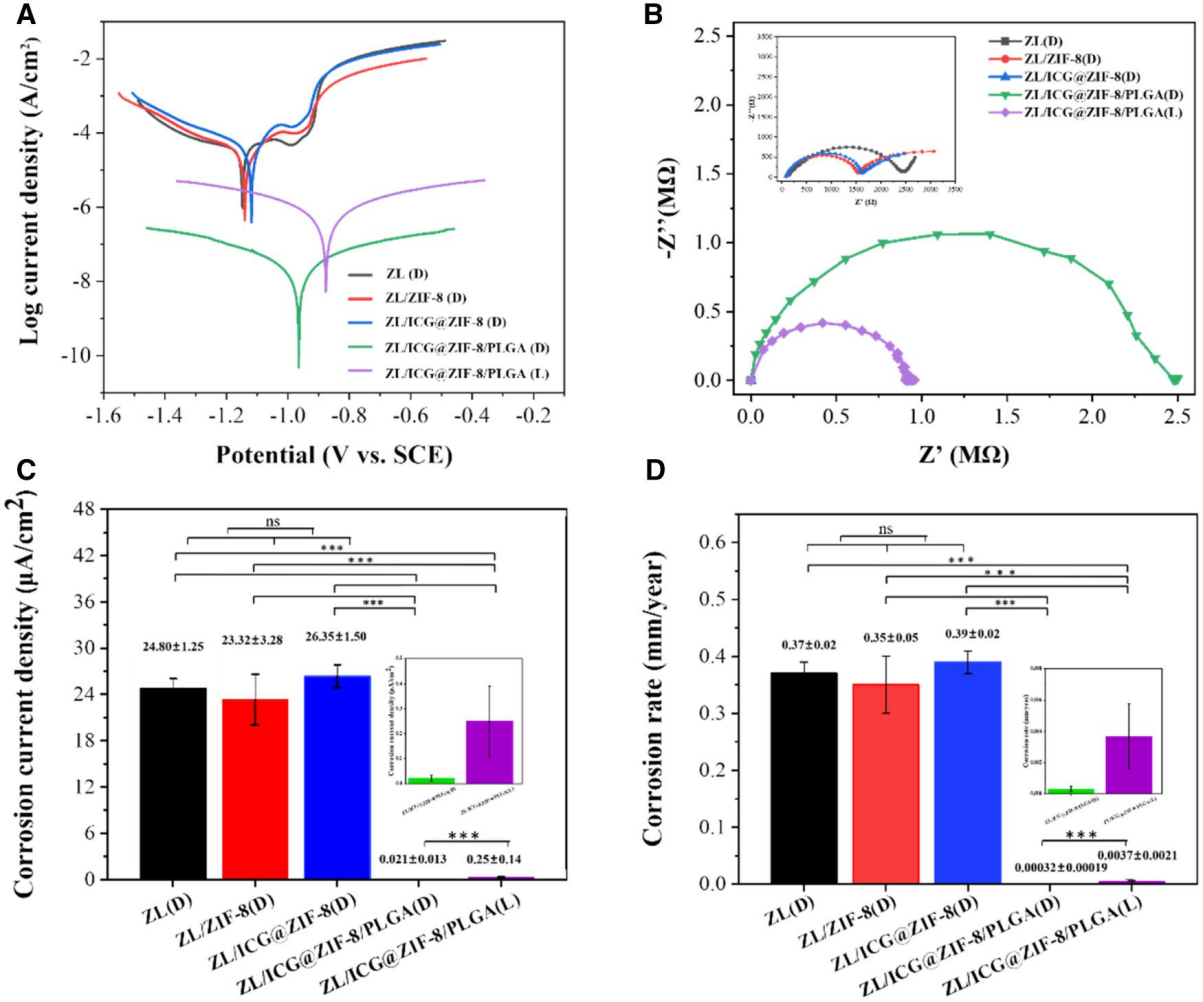
Electrochemical measurements of different samples in Hanks’ solution: (**A**) potentialdynamic curves; (**B**) Nyquist plots. Electrochemical metrics derived from tafel extrapolation include: (**C**) i_corr_ and (**D**) corrosion rate. A two-tailed student's t-test was used to statistically analyze the data (*P < 0.05, ** *P* < 0.01, *** *P* < 0.001).

Representative potential-dynamic polarization curves for all samples at 37°C Hanks’ solution were shown in Figure 3A. The relevant electrochemical metrics, including corrosion rate and corrosion current density (i_corr_) for all specimens, were determined through extrapolation (Figure 3C–D) [3, 31]. The i_corr_ values of the ZL alloy, ZL/ZIF-8 and ZL/ICG@ZIF-8/PLGA were (2.48 × 10^−5^), (2.33 × 10^−6^) and (2.10 × 10^−8^) A·cm^−2^, respectively. ZIF-8 and ICG@ZIF-8 layers had a minimal impact on corrosion resistance. However, the PLGA coating significantly reduced corrosion, dropping the current density from (2.48 × 10^−5^) to (2.10 × 10^−8^) A·cm^−2^.

In addition, the corrosion rate of ZL (0.37 mm/year) was similar to that of ZL coated with ZIF-8 (0.35 mm/year) and ZL coated with ICG@ZIF-8 (0.39 mm/year), and there was no significant change among the three. However, the corrosion rate for ZL (0.37 mm/year) was approximately three orders of magnitude greater than that for ZL/ICG@ZIF-8/PLGA (0.00032 mm/year). After exposure to light irradiation, the composite coating sustained damage, resulting in an increased i_corr_ of 2.50 × 10^−7^ A·cm^−2^, and an increased corrosion rate of 0.0037 mm/year. Compared with before light irradiation exposure, the i_corr_ and corrosion rate of ZL/ICG@ZIF-8/PLGA composite coating following NIR radiation were both increased. This suggested that the treated surface’s ability to withstand corrosion had decreased. These results showed that the PLGA layer had good corrosion resistance, ICG@ZIF-8/PLGA composite coating efficiently shielded the ZL alloy against electrolyte-induced corrosion, and NIR radiation significantly reduced the corrosion resistance of ZL/ICG@ZIF-8/PLGA.

Two capacitive and one inductive loops were visible in the ZL substrate spectrum’s Nyquist diagram (Figure 3B). The creation, adhesion and release of corrosion by-products were closely linked to the establishment of an induction circuit, indicating the dissolution of the metal matrix. The capacitive arc of ZIF-8 and ICG@ZIF-8 coatings was marginally lower than that of the ZL alloy. The capacitor arc of ICG@ZIF-8/PLGA group was significantly increased, which meant that PLGA coating could effectively prevent electrolyte penetration and reduce the Zn alloy’s rate of corrosion. However, the capacitor ring of ZL coated with ICG@ZIF-8/PLGA following NIR irradiation decreased obviously, but it was still higher than that of the uncoated Zn alloy, indicating that the coating’s ability to withstand corrosion declined after NIR light irradiation. The diameter of capacitive circuit directly affected the corrosion resistance of metal. Specifically, a smaller capacitor loop diameter indicated a quicker charge transfer rate on the metal’s surface. This increased rate of charge transfer could enhance the corrosion reaction. Consequently, the corrosion resistance of the metal was diminished. Conversely, a larger capacitive loop diameter might lead to a decrease in the corrosion reaction rate, thereby improving the metal’s ability to resist corrosion. This occurrence could be linked to the heat produced by the excited ICG within the coating, which raised the temperature of the PLGA to Tg. This increase in temperature promoted the mobility of the molecular chain segments, culminating in the creation of surface imperfections. Consequently, the electrolyte was able to infiltrate the coating through these gaps. This was consistent with the surface morphology characterization data. Consequently, with the exposure to NIR radiation, the photoreactive coating was able to efficiently achieve the distant manipulation of the degradation speed of the Zn alloy coating.

### Cytotoxicity assays

**Figure 4.**
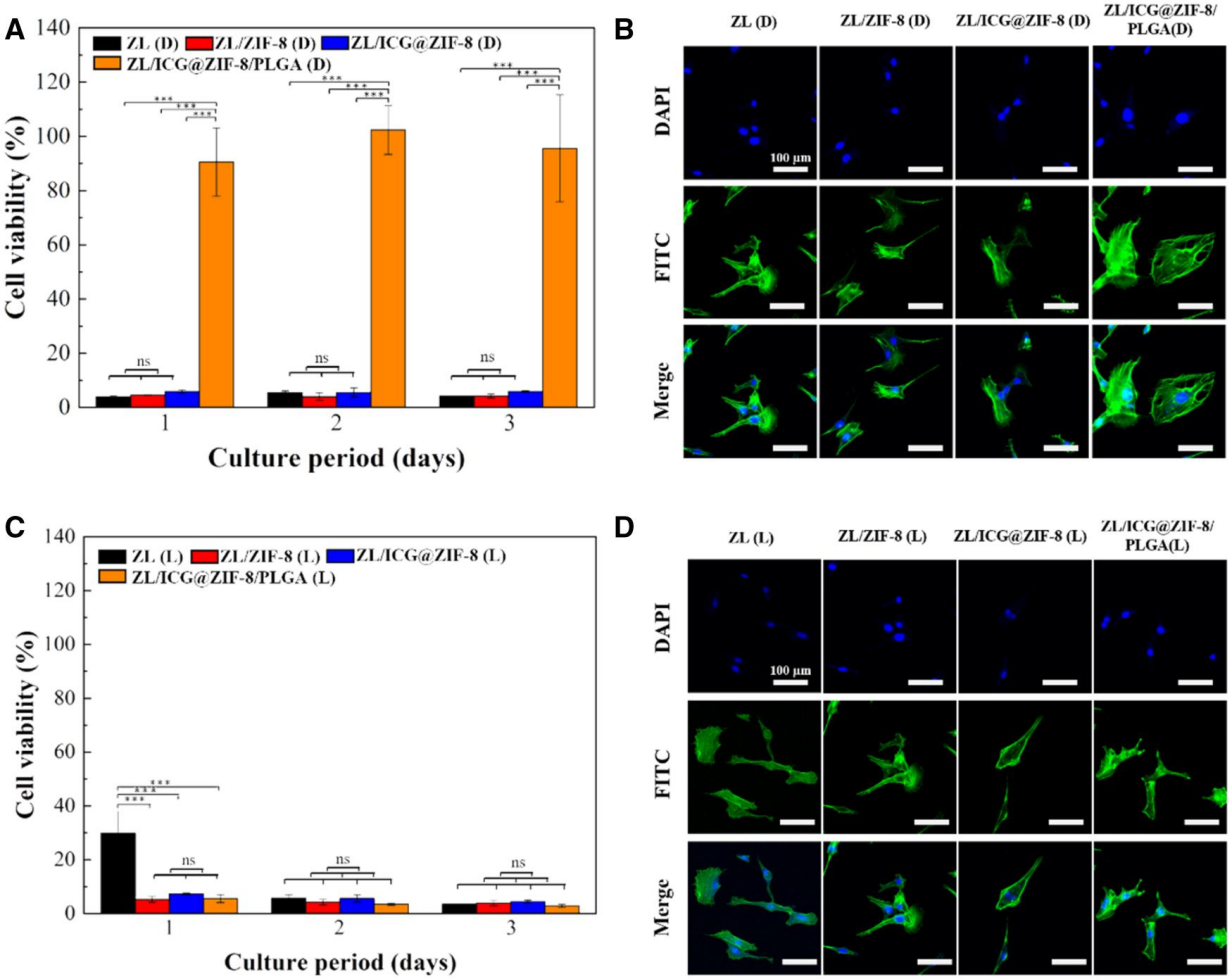
Assessment of the *in vitro* biocompatibility of different samples. (**A**) CCK-8 assays for MC3T3-E1 cells in the absence of light. (**B**) Fluorescent microscopy of MC3T3-E1 cells after 1 day of culture, with actin labeled by FITC (green) and nuclei by DAPI (blue), under dark conditions. (**C**) CCK-8 assays for MC3T3-E1 cells under 808-nm NIR light exposure. (**D**) Fluorescent microscopy of MC3T3-E1 cells after 1 day of culture, with actin labeled by FITC (green) and nuclei by DAPI (blue), under 808-nm NIR light exposure. A two-tailed student's t-test was used to establish statistical significance (*P < 0.05, **P < 0.01, ***P < 0.001).

Figure 4 showed the cell viability test results of different modified sample extracts cultured for 1, 2 and 3 days before and after exposing to NIR light at 808 nm. Extracts from ZL alloy, ZL/ZIF-8 and ZL/ICG@ZIF-8 showed no significant difference in cell viability before light irradiation exposure (Figure 4A). ZL/ICG@ZIF-8/PLGA exhibited good biocompatibility. PLGA in the hybrid coating effectively prevented substrate degradation and Zn^2+^ leakage and significantly improved the biocompatibility of samples [39, 40]. As shown in Figure 4C, following light irradiation, there was no discernible change between the test and control groups, and the coating extract had no significant effect on MC3T3-E1 viability. The cellular morphology observation (Figure 4B and D) further confined the quantitative results in Figure 4A and C. The results showed good biocompatibility of the ICG@ZIF-8/PLGA coating.

### *In vivo* osteogenesis experiments

**Figure 5.**
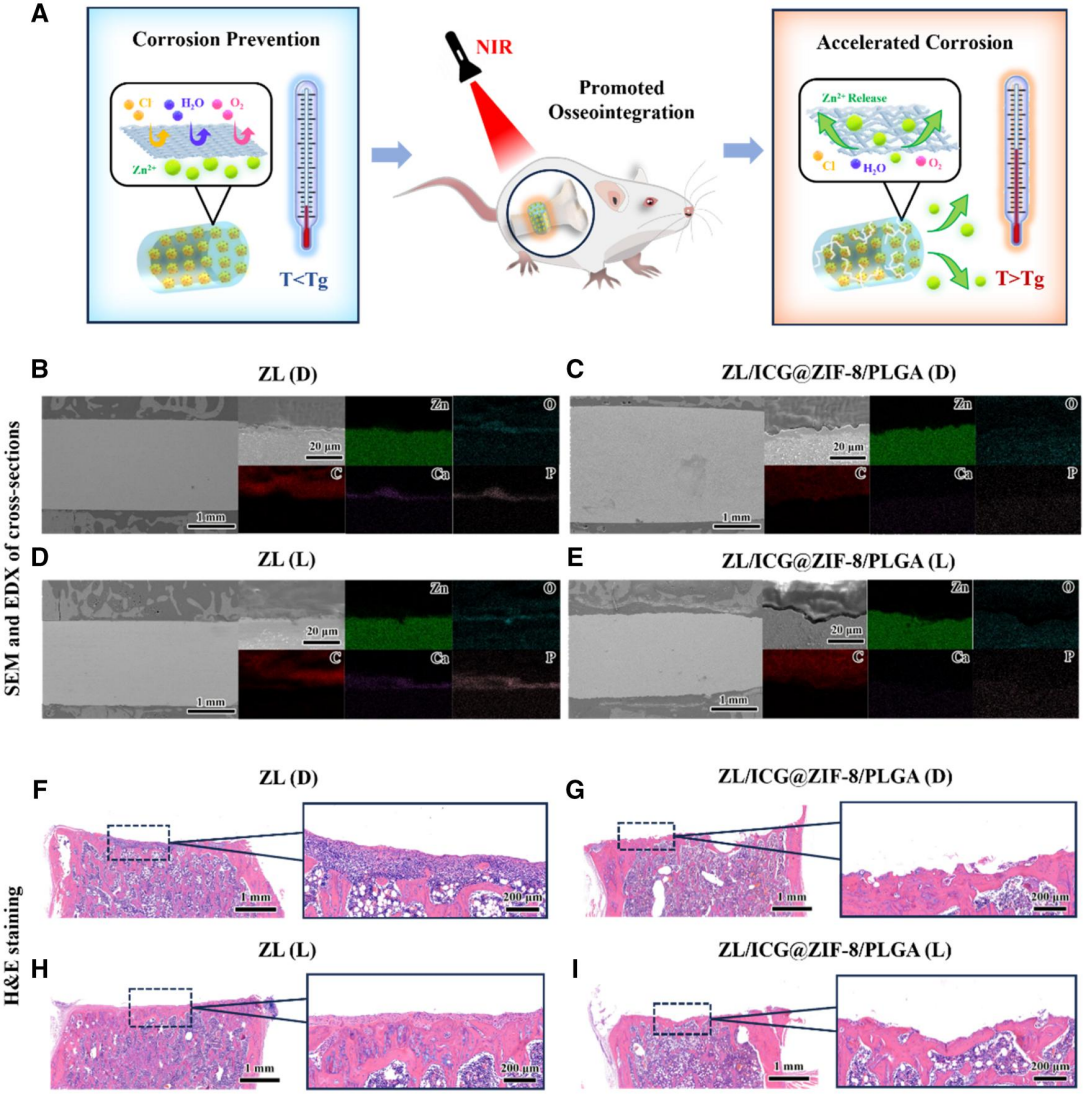
(**A**) Schematic diagram of mechanism and application of ZL/ICG@ZIF-8/PLGA photothermal osteogenesis *in vivo*. Cross-sectional SEM pictures of (**B**) ZL alloy, (**C**) ZL/ICG@ZIF-8/PLGA, (**D**) ZL alloy after NIR light irradiation and (**E**) ZL/ICG@ZIF-8/PLGA after NIR light irradiation for 8 weeks. Furthermore, the panels on the right-hand side showed the associated EDS mappings for the targeted elements within the delineated rectangular areas in (**B-E**). H&E staining of (**F**) ZL alloy, (**G**) ZL/ICG@ZIF-8/PLGA, (**H**) ZL alloy after NIR light irradiation and (**I**) ZL/ICG@ZIF-8/PLGA after NIR light irradiation for 2 months.

The mechanism and application of ZL/ICG@ZIF-8/PLGA photothermal osteogenesis *in vivo* were schematically depicted in Figure 5A. To examine the changes in the structure and makeup of the degradation, SEM and energy-dispersive spectroscopy (EDS) analyses were employed to examine the cross-sectional view of the implant-bone interface, as shown in Figure 5B–E. Only minor corrosion is visible, and there was no discernible difference in the corrosion of ZL in the body before and after NIR light irradiation. Two months following implantation, a layer of degradation products, primarily made up of Zn, O, C, Ca and P, covered the material’s surface. The peripheral part of the implant in the ZL group was occupied by new bone [34]. The corrosion of ZL was more severe than that of the samples modified without light, and after light, the corrosion of the modified group was between the above two. The by-products from the degradation of implantable biomaterials frequently exert a considerable influence on the surrounding tissue milieu. It was hypothesized that once PLGA degraded in a comparatively stable bone environment, the build-up of acidic degradation products would hasten the substrate’s rate of corrosion [35–37]. After exposure to light irradiation, the rate of PLGA breakdown rose. The degradation products were difficult to metabolize fast and collect locally, which lowered the local pH and hastens the substrate’s corrosion behavior.

According to H&E staining results (Figure 5F–I), the presence of inflammatory cells was noted in the ZL material before and after light irradiation exposure. There was little and loose braided bone surrounding the implant. Poor osseointegration resulted from a noticeable layer of fibrous connective tissue between the implant and the bone. In contrast, there were almost no inflammatory cells in the ZL/ICG@ZIF-8/PLGA group, and the local peripheral spacing was in intimate proximity to the neighboring bone tissue, suggesting a level of bone integration. In addition, no obvious tissue lesions, inflammation or injury was noted in the rat’s organs (heart, liver, lung, kidney) (Supplementary Appendix Figure 4). Therefore, PLGA had been shown to improve the inflammatory response of the implant and had a beneficial effect on new bone formation.

## Discussion

Zn alloy has recently gained attention as possible biomaterials. Nevertheless, existing studies indicate that the effects of Zn^2+^ on bone repair follow a dose-related pattern: low doses of Zn^2+^ stimulate the growth and differentiation of osteoblasts, while high doses lead to cytotoxicity and hindered bone fusion. At the initial phase of implant placement, due to tissue trauma, hematoma and inflammatory responses occur. At this point, the implant degrades relatively rapidly, releasing a large amount of Zn^2+^, hence the inhibitory effect of Zn^2+^ is predominant. During the later phases of implantation, once the *in vivo* conditions have normalized, the degradation benefit of the Zn alloy ought to be ideally reestablished. Considering the controlled corrosion rate based on the healing process of the injured tissue is promising. Given that the ion levels of degradation by-products are strongly correlated with the rate of corrosion, developing Zn-based materials with appropriate corrosion behavior is crucial.

**Figure 6.**
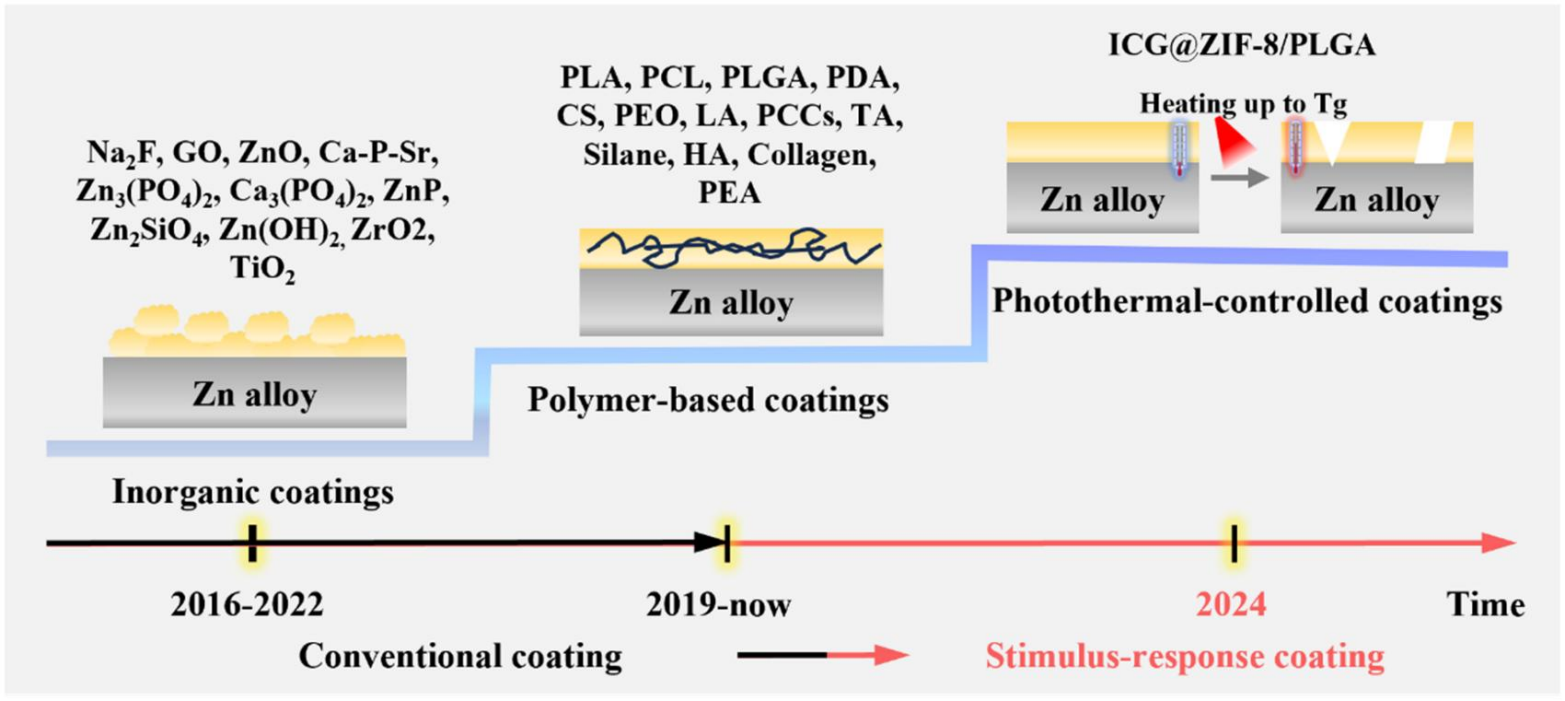
Methods of surface modification of Zn alloy: passive barrier to stimulus response [2, 11, 15–17, 42–44, 47–57].

Surface alteration is a powerful strategy to enhance the surface characteristics of Zn substrates. This is achieved through the establishment of a barrier that effectively separates the corrosive environment from direct contact with the substrate, thereby decelerating the corrosion dynamics. The application of the PEO process to coat pure Zn resulted in a coating with an inner barrier layer that demonstrated superior resistance performance. This is because the calcium phosphate-containing ZnO layer protects against corrosive ions [41]. The Zn alloy’s surface was coated with MAO-PLA [42], and the mixed coating effectively reduced the release of Zn^2+^ and had good biocompatibility. Coatings prepared on pure Zn exhibited 10-fold higher resistance to degradation compared to bare Zn alloy, where the PLA layer functioned as a robust barrier against the influx of corrosive ions [42]. A phenolic-copper amine cross-linked biodegradable coating, applied to the Zn alloy through a single-step electrophoretic deposition process, displayed a current density about 10 times less than the uncoated Zn alloy, indicating enhanced corrosion protection for the alloy. Additionally, after 30 days of degradation, the coating thickness reduced by approximately 5–7 µm while maintaining surface integrity, demonstrating long-term effective corrosion resistance [43]. However, existing coating methods mostly rely on the passive degradation of the coating itself, and the barrier effect may block the release of Zn^2+^, thus affecting the degradation advantages of degradable metal Zn alloys. Therefore, instead of completely blocking the release of Zn^2+^, the ideal coating is constructed to control the degradation rate and release Zn^2+^ appropriately (Figure 6) [2, 13, 15, 44–46].

In order to control the rate at which the coated ZL alloy degrades, we designed a photothermally responsive ICG@ZIF-8/PLGA biodegradable hybrid coating that was sensitive to 808-nm NIR radiation. In the initial stage of implantation, the PLGA protective layer prevented the implant from rapidly degrading and releasing a large amount of Zn^2+^ and enhanced the material’s biocompatibility. The photothermal action caused by the 808-nm NIR light raised the temperature of the PLGA coating to its Tg after the coating had completed its service purpose. This temperature increase facilitated the mobility of the PLGA molecular chain segments, accelerating the degradation of the coating and creating defects. These defects served as pathways for electrolyte penetration, enabling the underlying Zn alloy substrate to engage in reactions, thereby restoring the Zn alloy’s inherent corrosion resistance.

The electrochemical corrosion results showed that ZL coated with ICG@ZIF-8/PLGA hybrid coating had better anti-corrosion performance than ZL alloy. The ZL/ICG@ZIF-8/PLGA coating’s capacitor rings increased substantially, demonstrating that the PLGA layer acted as an efficient barrier to stop electrolyte reaching into the Zn alloy. This decreased the Zn alloy’s corrosion kinetics and stopped Zn^2+^ from being released. However, the capacitor ring of ZL/ICG@ZIF-8/PLGA coating decreased significantly under NIR light irradiation, but it remained superior to the uncoated Zn alloy, indicating that the coating’s corrosion resistance is diminished following NIR light exposure. The heat generated by the ICG components within the coating under NIR light irradiation could be responsible for raising the temperature of PLGA to its Tg, thereby enhancing the mobility of the molecular chain segments. Defects on the coated surface could arise from the mobility of this molecular chain component, allowing electrolytes to penetrate into the coating along these defects and then they react with the base metal. Therefore, it is crucial to control the degradation rates of Zn alloy to ensure a secure level of Zn^2+^ in the initial stage and restore the implant’s degradation after the coating has served its intended purpose. In this study, we can modify the pace of deterioration of Zn alloy to meet clinical requirements by regulating the irradiation duration and intensity.

Results indicated that the ICG@ZIF-8/PLGA coating had significantly improved cellular compatibility. NIR irradiation had accelerated the degradation of PLGA, restoring the release of Zn^2+^. Nevertheless, the diluted extract of this substance improved the MC3T3-E1 cells’ capacity for migration and viability (Supplementary Appendix Figures 2 and 3), presumably because the flowing body fluid had prevented local ion accumulation and ensured biocompatibility [58, 59]. Animal studies had shown that the coated group had exhibited good biocompatibility. Histological analysis supported the finding of no significant toxicity from Zn^2+^ and PLGA degradation products. Furthermore, blood tests and histological analyses indicated that the biosafety of Zn^2+^ was comparable to that of pure titanium [60]. PLGA was known for its good biocompatibility and was metabolized into CO_2_ and H_2_O in the body, and based on PLGA commercial products, such as absorbable sutures, drug delivery platforms, bone screws, etc, it has been widely used in orthopedics [56, 43]. In summary, the degradation by-products of Zn^2+^ and PLGA do not pose significant risks for tissue biocompatibility.

Overall, initially, with the barrier effect of the dense PLGA polymer coating, ZL/ICG@ZIF-8/PLGA exhibited excellent corrosion resistance, hindering excessive leakage of Zn^2+^ to improve the biocompatibility of Zn alloy. NIR could be regarded as means of transition from corrosion resistance to active degradation for Zn alloys. When NIR was activated, the temperature of PLGA gradually rose to the Tg, promoting the transformation of PLGA into a viscous state, expanding the intermolecular gaps and resulting in shape deformation. This directly facilitated the penetration of external electrolytes, which reacted directly with the Zn substrate, thus significantly restoring the Zn alloy’s inherent corrosion resistance. Therefore, NIR-controlled corrosion of Zn alloys can be achieved, meeting the demand for controllable degradation rates.

## Conclusion

In this work, ICG@ZIF-8/PLGA photoresponsive composite coatings were constructed on Zn alloys to control biocorrosion for orthopedic applications. The mechanism was that the ICG components in the coating are activated to cause hyperthermia when exposed to 808-nm NIR light, heating the PLGA to its Tg, resulting in molecular segment displacement, and ultimately resulting in coating defects. As a result, the electrolyte was able to travel through the coating via these imperfections and interact with the Zn alloy, restoring the Zn alloy’s inherent corrosion resistance. In addition, the hybrid coating displayed acceptable biocompatibility *in vitro* and *in vivo*. Taken together, this NIR-sensitive coating improved the biocompatibility of Zn alloy and presented a promising tool for regulating its degradation behavior.

## Supplementary Material

rbaf001_Supplementary_Data
