# Supplementary material for: Photothermally controlled ICG@ZIF-8/PLGA coating to modify the degradation behavior and biocompatibility of Zn-Li alloy for bone implants
Source: Regen Biomater. 2025 Jan 6;12:rbaf001. doi: 10.1093/rb/rbaf001 (PMC11879299; doi:10.1093/rb/rbaf001)
Supplement: rbaf001_Supplementary_Data [file rbaf001_supplementary_data.zip › bc3b9_Supplementary_File_for_Review.docx]

Appendix

Photothermally controlled ICG@ZIF-8/PLGA coating to modify the degradation behavior and biocompatibility of Zn-Li alloy for bone implants

Ting Zhang ^a, b,1^, Yameng Yu ^b,1^, Wei Yuan ^b^, Zeqi Ren ^b^, Yan Cheng ^b^, Shuilin Wu ^b^***, Yufeng Zheng ^b^**, Dandan Xia ^a^*

^a^ Department of Dental Materials, Peking University School and Hospital of Stomatology & National Center for Stomatology & National Clinical Research Center for Oral Diseases & National Engineering Research Center of Oral Biomaterials and Digital Medical Devices& Beijing Key Laboratory of Digital Stomatology & NHC Key Laboratory of Digital Stomatology & NMPA Key Laboratory for Dental Materials, Beijing 100081, China

^b^ School of Materials Science and Engineering, Peking University, Beijing 100871, China

^1^ Authors contributing equally to this article and joint first authors

* Corresponding author:

Prof. Dandan Xia

Department of Dental Materials, Peking University School and Hospital of Stomatology, No.22, Zhongguancun South Avenue, Haidian District, Beijing 100081, China

E-mail: [dandanxia@pku.edu.cn](mailto:liuyunsong@hsc.pku.edu.cn)

** Corresponding author:

Prof. Yufeng Zheng

School of Materials Science and Engineering, Peking University, No.5 Yi-He-Yuan Road, HaiDian District, Beijing 100871, China

E-mail: [yfzheng@pku.edu.cn](mailto:yfzheng@pku.edu.cn)

*** Corresponding author:

Prof. Shuilin Wu

School of Materials Science and Engineering, Peking University, No.5 Yi-He-Yuan Road, HaiDian District, Beijing 100871, China

*Email: [slwu@pku.edu.cn](mailto:slwu@pku.edu.cn)

Results


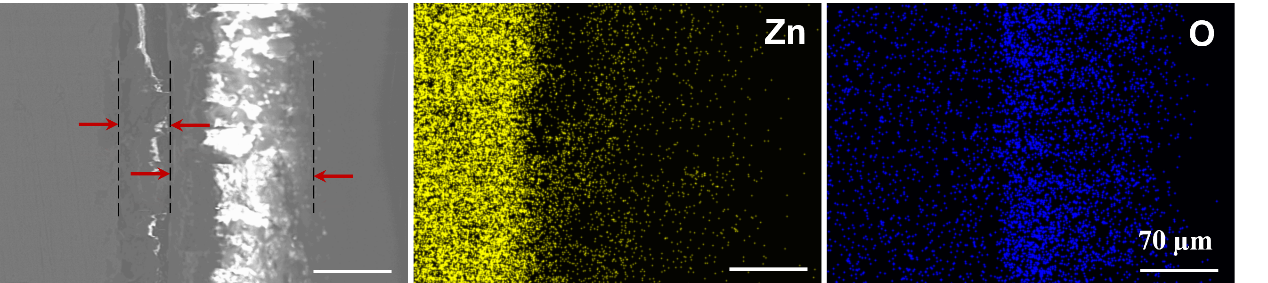


**Appendix Figure 1**. The thickness of ZL/ICG@ZIF-8/PLGA coating.


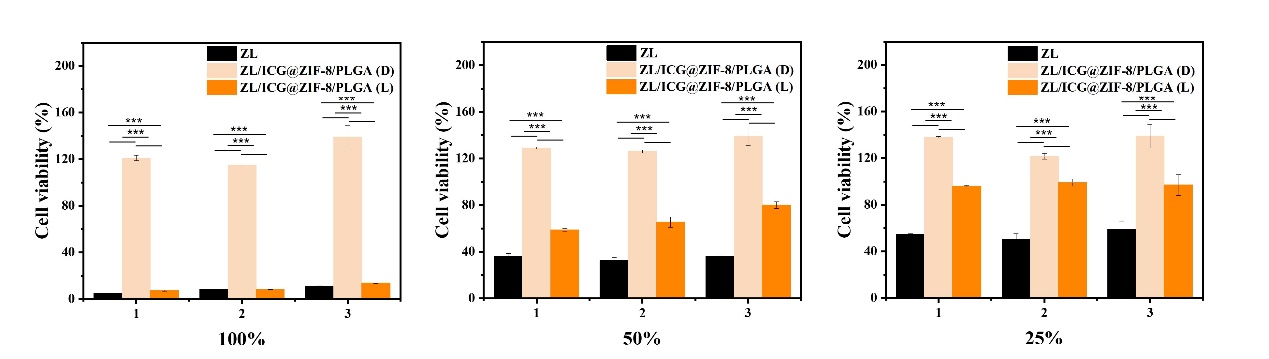


**Appendix Figure 2**.CCK-8 tests of MC3T3-E1 cells cultured in extracts from each sample for 1, 2 and 3 days of different concentrations of extracts of ZL, ZL/ICG@ZIF-8/PLGA without illumination and ZL/ICG@ZIF-8/PLGA after NIR light irradiation *in vitro*.


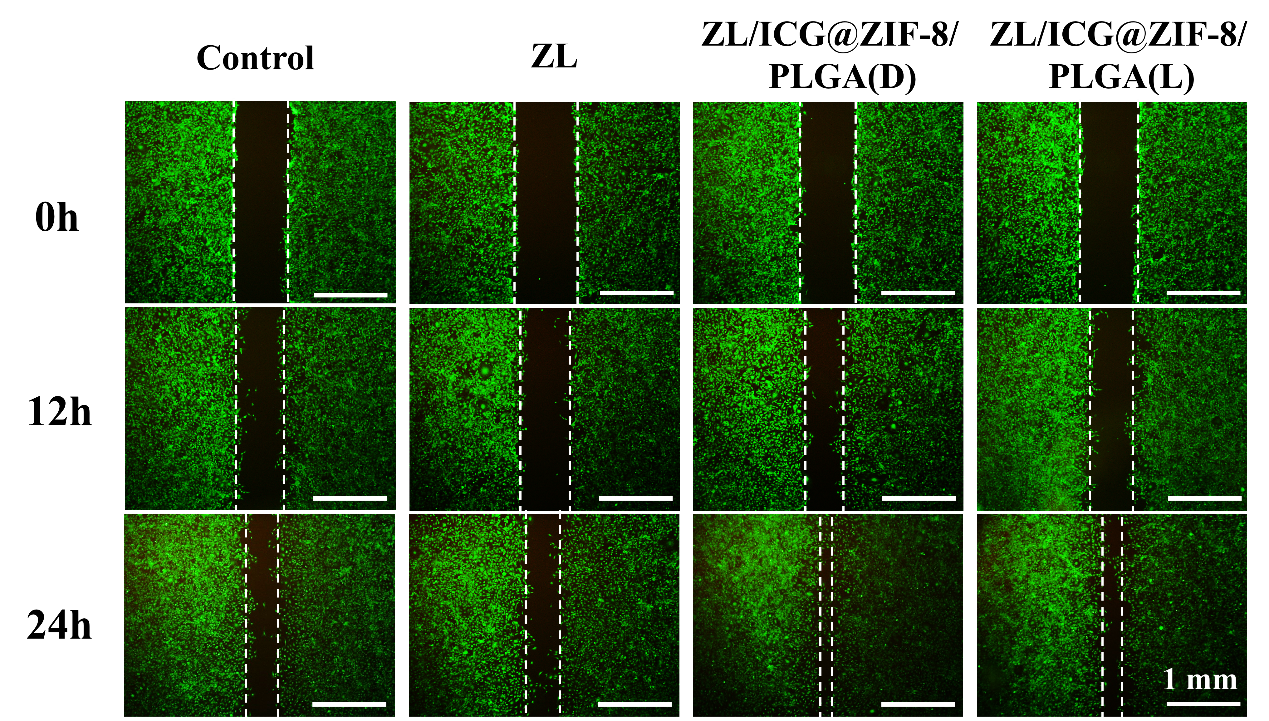


**Appendix Figure 3**. Cell migration of MC3T3-E1 cells in wound healing tests of ZL, ZL/ICG@ZIF-8/PLGA without illumination and ZL/ICG@ZIF-8/PLGA after NIR light irradiation with 25% concentrations of extracts *in vitro*.


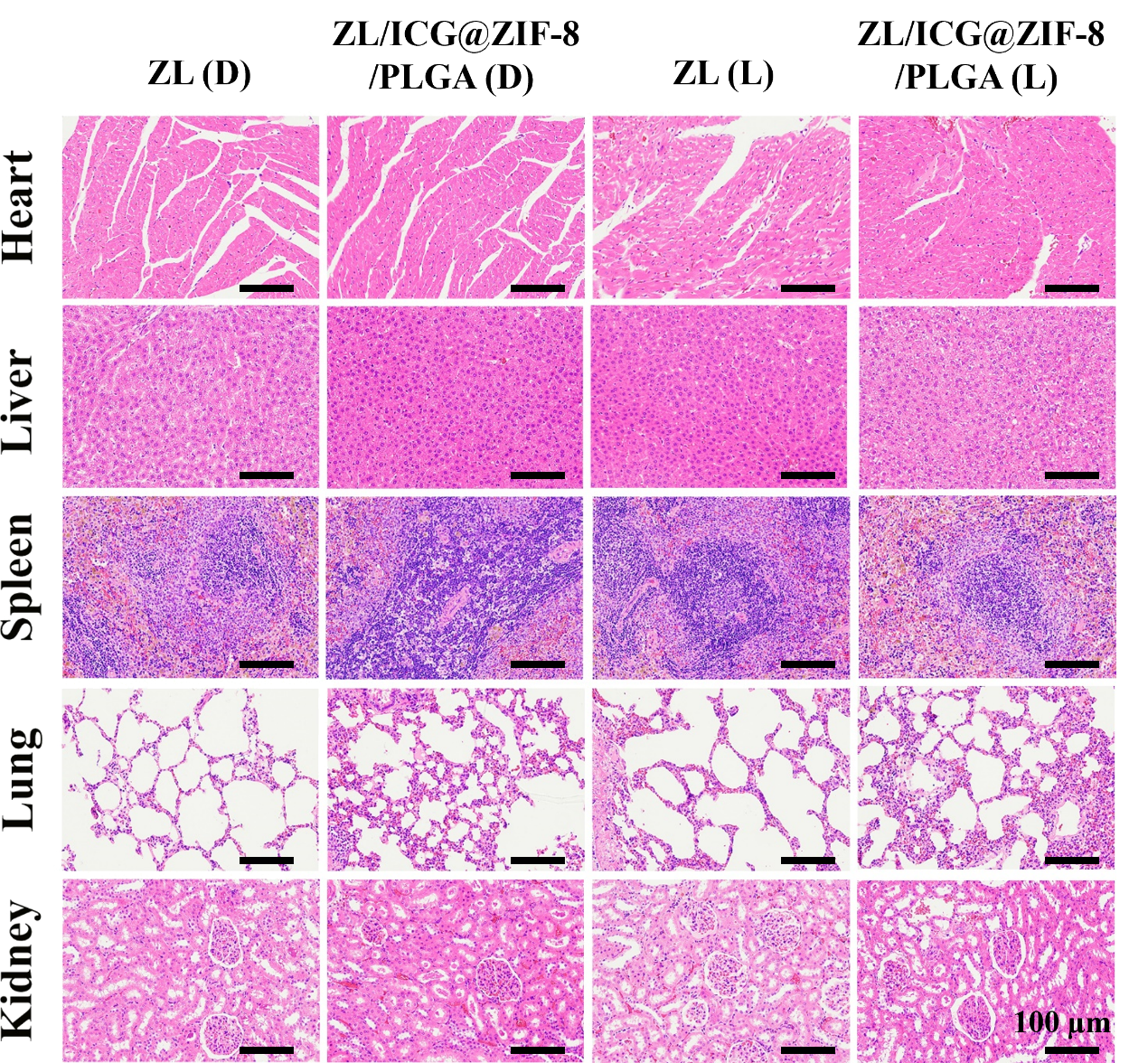


**Appendix Figure 4**. Histological images of diverse organs after 8 weeks of implantation of ZL alloy, ZL/ICG@ZIF-8/PLGA coating, ZL alloy after NIR light irradiation and ZL/ICG@ZIF-8/PLGA coating after NIR light irradiation.
